# The QUIC-SP: A Spanish language tool assessing unpredictability in early life is linked to physical and mental health

**DOI:** 10.1371/journal.pone.0298296

**Published:** 2025-01-24

**Authors:** Sabrina R. Liu, Natasha A. Bailey, Sara Romero-González, Amy Moors, Belinda Campos, Elysia Poggi Davis, Laura M. Glynn

**Affiliations:** 1 Department of Psychology, Crean College of Health and Behavioral Sciences, Chapman University, Orange, California, United States of America; 2 Department of Chicano/Latino Studies, University of California-Irvine, Irvine, California, United States of America; 3 Department of Psychology, University of Denver, Denver, Colorado, United States of America; 4 Department of Pediatrics, University of California-Irvine, Irvine, California, United States of America; Yarmouk University, JORDAN

## Abstract

Accumulating evidence indicates that unpredictable signals in early life represent a unique form of adverse childhood experiences (ACEs) associated with disrupted neurodevelopmental trajectories in children and adolescents. The Questionnaire of Unpredictability in Childhood (QUIC) was developed to assess early life unpredictability [1], encompassing social, emotional, and physical unpredictability in a child’s environment, and has been validated in three independent cohorts. However, the importance of identifying ACEs in diverse populations, including non-English speaking groups, necessitates translation of the QUIC. The current study aims to translate and validate a Spanish language version of the QUIC (QUIC-SP) and assess its associations with mental and physical health. Spanish-speaking participants (*N* = 285) were recruited via the online market crowdsourcing platform, Amazon Mechanical Turk (MTurk), and completed an online survey that included the QUIC-SP and validated Spanish language assessments of physical and mental health. The QUIC-SP demonstrated excellent psychometric properties and similar mean scores, endorsement rates, and internal reliability to the English language version, thus establishing its validity among Spanish-speaking adults. Higher QUIC-SP scores, indicating greater unpredictability in early life, predicted increased symptoms of anxiety, anhedonia, depression, and poorer physical health. Given significant racial and ethnic disparities in health, the QUIC-SP may serve as a valuable tool to address the public health consequences of ACEs among Spanish-speaking populations.

## Introduction

A substantial body of work has established that adverse childhood experiences (ACEs) negatively impact physical and mental health across the lifespan through mechanisms related to neurodevelopmental disruption, alterations in stress, metabolic and immune systems, as well as epigenetic modifications [2–5]. However, within this body of research, what has been conceptualized as “adversity” greatly varies and some forms have been studied more than others (e.g., poverty, parental separation, abuse). Unpredictable signals (e.g., social, emotional, and physical unpredictability) from caregivers and the environment represent a novel conceptualization of an additional ACE [6–11]. The importance of unpredictability of sensory inputs was initially supported by preclinical work demonstrating that unpredictable maternal signals (patterns of licking, grooming, and nursing in rodents) causally influence the maturation of brain systems that govern emotional and cognitive functions [12–15]. Further validating the importance of unpredictability in childhood, several independent human cohorts have confirmed that unpredictable environmental signals contribute to adverse neurodevelopmental outcomes and compromised mental health, even after accounting for the predictive capacity of previously established ACEs [1]. To date, exposures to unpredictable caregiver(s) signals in infancy have been linked to trajectories of cognitive and emotional functioning through adolescence [7, 16–19]. These findings across species warrant additional research among diverse populations on unpredictability as a novel type of early life adversity as a step to address the significant and costly public health consequences of ACEs.

Given that unpredictability in early life occurs in a broad range of contexts (e.g., parental, family, neighborhood) and in different distributions over time (e.g., from seconds to years), we recognized the need to develop a comprehensive self-report measure. To address this unmet need, the Questionnaire of Unpredictability in Childhood (QUIC) was developed [1]. This 38-item measure captures aspects of the early environment that are characterized by stochastic variation in events or exposures and that cannot be anticipated or foreseen with a high degree of certainty. In the validation paper, we show that the QUIC demonstrated excellent psychometrics (internal consistency, test-retest reliability) and associations with mental health (depression, anhedonia, and anxiety) in three independent cohorts: adult females, male military veterans, and adolescents. Further, the QUIC’s contribution to mental health persisted above and beyond other established ACE predictors, such as parenting, income-to-needs ratio, traumatic life events, and child maltreatment. Importantly, research has demonstrated accuracy of self-reports on the QUIC. We found agreement between specific QUIC scale items (e.g., moving frequently) and longitudinal data that was collected from birth in a cohort of adolescents. Additionally, prospective measures of unpredictability assessed during infancy and childhood were associated with QUIC scores obtained in adolescence [1].

As the first comprehensive self-report measure of unpredictability in childhood, the QUIC enables researchers and clinicians to easily gather information about a unique predictor of compromised health and development. An important next step is to ensure this measure is widely accessible, which includes translation into other languages. Although researchers have highlighted heightened risk of early adversity to be a potential root cause of existing racial/ethnic disparities in health and development [20], these topics have been less studied in culturally diverse populations. Hispanic/Latinos (for use of these terms see [21]) make up the second largest racial/ethnic group in the U.S. (after whites), and Spanish constitutes the second most spoken language with over 41 million speakers aged five and older [22–24]. Thus, to better understand the impact of early childhood experiences, including unpredictability, and to meet the needs of diverse Hispanic/Latino populations, the goals of the current study were to: 1) translate and validate a Spanish language version of the QUIC (QUIC-SP), and 2) examine how unpredictability in early childhood (via the newly translated QUIC-SP measure) is associated with mental and physical health outcomes. We expected that the QUIC-SP would demonstrate negative associations with mental health akin to those found by Glynn and colleagues with the English-language QUIC. Although not previously explored with the QUIC, it was also expected that the QUIC-SP would have an inverse relation to physical health, consistent with previous research on other ACE types [25, 26].

## Methods

### Description of scale

The Questionnaire of Unpredictability in Childhood (QUIC) is a self-report measure designed to assess unpredictability in social, emotional and physical domains of the childhood environment. Details regarding the development of the original English language version of the scale can be found in Glynn et al. (2019).

The English language version of the QUIC is intended for people aged 12 and older, as the 38 items assess family and home conditions prior to age 18, with a subset of items focusing on life circumstances prior to age 12. It comprises five subscales: Parental Monitoring and Involvement (parent kept track of child’s activities; 9 items), Parental Predictability (stability and presence of a parent; 12 items), Parental Environment (stability in parent’s life; 7 items), Physical Environment (household chaos, moving; 7 items), and Safety and Security (fear of lacking basic necessities; 3 items). Respondents answer yes (1) or no (0) for each item. Total scores range from 0 to 38, with higher scores representing greater childhood unpredictability. The scale exhibits excellent test-retest reliability (r = .92) and internal consistency (α = .89). The validity of the measure is supported by the fact that QUIC scores are positively associated with prospectively measured indicators of unpredictability in infancy and childhood (unpredictability of maternal mood and sensory signals) and that accuracy of recall was confirmed with prospective data (e.g., number of household moves, parental separation) [1].

### Translation

First, the QUIC was translated into Spanish by an English-Spanish bilingual individual, whose childhood was spent in both Mexico and the U.S. After completing the initial translation, the translator reviewed and discussed the items with five bilingual individuals for cultural relevance and interpretability, including an English-Spanish bilingual psychologist and expert in Latino culture, a Spanish teacher living in Mexico, and three native Spanish speakers who reside in the U.S. This approach to receiving feedback from individuals who were formal educators of Spanish and community members who spoke Spanish helped ensure accessible translation of items for a wide range of Spanish speakers with different backgrounds [27]. After finalizing the items, the scale was then back-translated by a sixth, independent, bilingual English-Spanish speaker to confirm the quality and integrity of the translation. The English and Spanish versions of the items can be found in Table 1.

**Table 1:** Questionnaire of Unpredictability in Childhood (QUIC) items by subscale in Spanish and English.

Spanish	English
**Supervisión Parental e Involucración**	**Parental Monitoring and Involvement**
Hasta la edad de 12 años: Tenía una rutina mañanera establecida en los días escolares (por ejemplo, normalmente hacía lo mismo cada día para prepararme).(R)	Prior to age 12: I had a set morning routine on school days (i.e., I usually did the same thing each day to get ready).(R)
Hasta la edad de 12 años: Mis padres estaban pendientes de lo que comía (por ejemplo, se aseguraban de que no me faltara una comida o intentaban asegurarse de que comiera saludable).(R)	Prior to age 12: My parents kept track of what I ate (e.g., made sure that I didn’t skip meals or tried to make sure I ate healthy food).(R)
Hasta la edad de 12 años: La mayoría de los días comíamos en familia.(R)	Prior to age 12: My family ate a meal together most days (R)
Hasta la edad de 12 años: Mis padres intentaban asegurarse de que durmiera bien por la noche (por ejemplo, tenía un horario regular de ir a dormir, mis padres revisaban que estuviera dormido).(R)	Prior to age 12: My parents tried to make sure I got a good night’s sleep (e.g., I had a regular bedtime, my parents checked to make sure I went to sleep).(R)
Hasta la edad de 12 años: Tenía una rutina antes de acostarme a dormir (por ejemplo, mis padres me cobijaban, me leían un libro, yo tomaba un baño).(R)	Prior to age 12: I had a bedtime routine (e.g., my parents tucked me in, my parents read me a book, I took a bath).(R)
Hasta la edad de 12 años: Al menos uno de mis padres sabía lo que yo hacía en mis horas después de escuela o en mi tiempo libre.(R)	Prior to age 12: In my afterschool or free time hours at least one of my parents knew what I was doing.(R)
Hasta la edad de 12 años: Al menos uno de mis padres regularmente revisaba que yo hiciera mi tarea.(R)	Prior to age 12: At least one of my parents regularly checked that I did my homework.(R)
Hasta la edad de 18 años: Al menos uno de mis padres regularmente estaba pendiente de mi progreso en la escuela.(R)	Prior to age 18: At least one of my parents regularly kept track of my school progress.(R)
Hasta la edad de 18 años: Al menos uno de mis padres hacía tiempo para ver cómo yo estaba todos los días.(R)	Prior to age 18: At least one parent made time each day to see how I was doing.(R)
**Previsibilidad Parental**	**Parental Predictability**
Hasta la edad de 12 años: Muchas veces mis padres llegaban tarde a recogerme (por ejemplo, de la escuela, del cuidado después de escuela o de deportes).	Prior to age 12: My parents were often late to pick me up (e.g. from school, aftercare or sports).
Hasta la edad de 12 años: Normalmente sabía cuándo mis padres iban a estar en casa.(R)	Prior to age 12: I usually knew when my parents were going to be home.(R)
Hasta la edad de 18 años: Al menos uno de mis padres tenía castigos que eran impredecibles.	Prior to age 18: At least one of my parents had punishments that were unpredictable.
Hasta la edad de 18 años: Muchas veces me preguntaba si alguno de mis padres volvería a casa al final del día.	Prior to age 18: I often wondered whether or not one of my parents would come home at the end of the day.
Hasta la edad de 18 años: Mi familia planeaba actividades para hacer juntos.(R)	Prior to age 18: My family planned activities to do together.(R)
Hasta la edad de 18 años: Al menos uno de mis padres planeaba algo para la familia pero después no llevaba el plan a cabo.	Prior to age 18: At least one of my parents would plan something for the family, but then not follow through with the plan.
Hasta la edad de 18 años: Mi familia tenía tradiciones para los días festivos que hacíamos todos los años (por ejemplo, cocinando comida especial a cierta época del año, decorar la casa de la misma manera).(R)	Prior to age 18: My family had holiday traditions that we did every year (e.g., cooking a special food at a particular time of year/decorate the house the same way).(R)
Hasta la edad de 18 años: Al menos uno de mis padres era desorganizado.	Prior to age 18: At least one of my parents was disorganized.
Hasta la edad de 18 años: Al menos uno de mis padres era impredecible.	Prior to age 18: At least one of my parents was unpredictable.
Hasta la edad de 18 años: Para al menos uno de mis padres, cuando ellos estaban molestos, no sabía cómo ellos iban a actuar.	Prior to age 18: For at least one of my parents, when they were upset I did not know how they would act.
Hasta la edad de 18 años: Uno de mis padres podría pasar en un instante de la calma a la furia.	Prior to age 18: One of my parents could go from calm to furious in an instant.
Hasta la edad de 18 años: Uno de mis padres podría pasar en un instante de la calma al estrés y los nervios.	Prior to age 18: One of my parents could go from calm to stressed or nervous in an instant.
**Entorno Parental**	**Parental Environment**
Hasta la edad de 18 años: Hubo un largo periodo de tiempo cuando no vi a uno de mis padres (por ejemplo, despliegue militar, tiempo en la cárcel, acuerdo de mi custodia).	Prior to age 18: There was a long period of time when I didn’t see one of my parents (e.g. military deployment, jail time, custody arrangements).
Hasta la edad de 18 años: Experimenté cambios en el acuerdo de mi custodia.	Prior to age 18: I experienced changes in my custody arrangement.
Hasta la edad de 18 años: Al menos uno de mis padres frecuentemente cambiaba de trabajo.	Prior to age 18: At least one of my parents changed jobs frequently.
Hasta la edad de 18 años: Hubo tiempos cuando uno de mis padres estaba desempleado y no podía encontrar trabajo aunque él/ella quería.	Prior to age 18: There were times when one of my parents was unemployed and couldn’t find a job even though he/she wanted one.
Hasta la edad de 18 años: Mis padres tenían una relación estable entre ellos. (R)	Prior to age 18: My parents had a stable relationship with each other.(R)
Hasta la edad de 18 años: Mis padres se divorciaron.	Prior to age 18: My parents got divorced.
Hasta la edad de 18 años: Al menos uno de mis padres tenía muchas parejas románticas.	Prior to age 18: At least one of my parents had many romantic partners.
**Entorno Físico**	**Physical Environment**
Hasta la edad de 18 años: Muchas veces había gente entrando y saliendo de mi casa que yo no me esperaba que estuvieran ahí.	Prior to age 18: There were often people coming and going in my house that I did not expect to be there.
Hasta la edad de 18 años: Me mudé frecuentemente.	Prior to age 18: I moved frequently.
Hasta la edad de 18 años: Cambié frecuentemente de escuelas.	Prior to age 18: I changed schools frequently.
Hasta la edad de 18 años: Cambié de escuelas a mitad del año.	Prior to age 18: I changed schools mid-year.
Hasta la edad de 18 años: Vivía en una casa limpia.(R)	Prior to age 18: I lived in a clean house.(R)
Hasta la edad de 18 años: Vivía en una casa desordenada (por ejemplo, montones de cosas por todos lados).	Prior to age 18: I lived in a cluttered house (e.g., piles of stuff everywhere).
Hasta la edad de 18 años: En mi casa, las cosas que necesitaba muchas veces no estaban en su lugar, y no las podía encontrar.	Prior to age 18: In my house things I needed were often misplaced so that I could not find them.
**Seguridad y Protección**	**Safety and Security**
Hasta la edad de 18 años: Hubo un periodo de tiempo en el que muchas veces me preocupaba de no tener suficiente comida para comer.	Prior to age 18: There was a period of time when I often worried that I was not going to have enough food to eat.
Hasta la edad de 18 años: Hubo un periodo de tiempo en el que muchas veces me preocupaba que mi familia no tuviera suficiente dinero para pagar por necesidades como ropa o pagos.	Prior to age 18: There was a period of time when I often worried that my family would not have enough money to pay for necessities like clothing or bills.
Hasta la edad de 18 años: Hubo un periodo de tiempo en el que no me sentí seguro en mi hogar.	Prior to age 18: There was a period of time when I did not feel safe in my home.

*Note*. R indicates reverse scored item. Respondents answer yes (1) or no (0) for each item.

### Participants and procedures

The study was approved by the Chapman University Institutional Review Board. The recruitment period for the study began on November 24, 2020 and ended on November 28, 2020. Participants were recruited through Amazon Mechanical Turk (MTurk), an online market crowdsourcing platform where users can complete surveys for monetary incentives. Inclusion criteria consisted of the following: the participant must speak and read Spanish fluently, be 18 years of age or older, and live in the United States. MTurk users who met eligibility criteria were able to view the description of the survey and could access the survey via a link, which was administered through the Research Electronic Data Capture (REDCap) online survey platform. Participants were then presented with the written electronic informed consent form. If they consented to participate, they proceeded to the main survey.

A total of 498 respondents consented to participate in the survey that included the QUIC, demographic information, and measures of mental and physical health. Ninety-three participants were excluded for not completing the survey and 120 participants were excluded for failing one or more of the three attention checks, resulting in a final count of 285 participants (57.9% female). The attention checks consisted of items embedded within measures asking participants to select a particular response for that item (e.g., “please select ‘totally disagree’ for this question”). Importantly, inattentiveness and high rates of attrition on online surveys is common, with previous research documenting estimates as high as 60% of respondents answering items carelessly [28, 29]. However, compared to research using different recruitment strategies (e.g., participant pools), research has suggested MTurk respondents are more attentive to instructions and items [28]. Excluding responses from those who fail manipulation checks ensures MTurk survey data is high-quality and reliable [30–32]. Moreover, the MTurk platform has been shown to be a reliable way to recruit Spanish-speaking participants and has been used in clinical and non-clinical scale validation research [33–35]. Demographic information of the final study sample is reported in Table 2.

**Table 2:** Descriptive statistics.

	*N* (%)
**Age**	
18–24 years	19.6
25–34 years	38.9
35–44 years	25.6
45–54 years	9.5
55–64 years	5.3
65+ years	1.1
**Gender**	
Female	57.9
Male	41.4
Transgender	0.4
Non-binary	0.4
**Race/ethnicity**	
Asian	3.5
Black/African American	2.1
Hispanic, Latino or Spanish	67
White/Caucasian	27
Other	0.4
**Highest level of education**	
Less than 6 years	7.7
6–10 years	6.7
11–15 years	26
16–20 years	53.5
21–25 years	5.7
More than 25 years	0.7
**Annual household income**	
$5,000-$34,999	26.8
$35,000-$64,999	36.9
$65,000-$99,999	20.4
$100,000-$249,999	14.8
$250,000-$449,999	1.5
**Nativity**	
United States	77.5
Mexico	6.7
Central America	2.1
South America	8.8
Europe	2.5
Other	2.5
**Native Language**	
Spanish	50.9
English	49.1

### Measures

**Mental health assessment.** *Depressive symptoms*. Depressive symptoms were assessed with the Spanish version of the Beck Depression Inventory-II (BDI-II; [36]). The BDI-II is a 21-item instrument that is valid for use in both psychiatric and non-psychiatric populations [37]. The measure assesses 21 depressive symptoms rated on a 0–3 scale, with higher scores indicating greater symptom severity. Internal consistency was excellent in the current sample (α = .94).

*Anxiety*. Symptoms of anxiety were measured with a 9-item version of the State Anxiety Scale from the State-Trait Anxiety Inventory (STAI; [38]) that has been adapted for use in Spanish-speaking populations [39]. Participants rated their agreement with 9 statements about anxiety symptoms experienced at the time of assessment and in the days leading up to assessment (e.g., “I am worried”) on a 4-point Likert scale from 1 (not at all) to 4 (very much). It is important to note that the original STAI contains 10 items; however in this survey, one item was excluded by mistake. Despite this error, internal consistency for the current sample was very high at .90, thus providing support to the validity of the 9-item STAI utilized in this study. As an additional validity check, we imputed values for the missing item and found no significant changes in the overall pattern of results.

*Anhedonia*. Anhedonia was examined using the 14-item Snaith-Hamilton Pleasure Scale (SHAPS; [40]). The Spanish version of the scale has been found to have good internal consistency and construct validity [41]. Participants were asked to indicate how much they enjoyed various pleasurable experiences (e.g., “I would enjoy a warm bath or refreshing shower”). Responses were recorded on a 4-point Likert scale from 1 (strongly disagree) to 4 (strongly agree). Internal consistency for the SHAPS in the current sample was .86.

**Physical health assessment.** *Physical functioning*. Assessment of physical functioning was measured using the Spanish version of the Patient-Reported Outcomes Measurement Information System (PROMIS–57) Physical Functioning subscale [42]. This subscale contains 8 items; participants rated how difficult it was for them to complete various physical activities (e.g., “Are you able to go for a walk of at least 15 minutes?”) using a 5-point scale from 1 (unable to do) to 5 (without difficulty). Internal consistency in the current sample was high (α = .93).

*Global health*. Participants’ global health was assessed using one question from the Spanish version of the Health-Related Quality of Life (HRQOL) scale [43]. Participants were asked to give a general rating of their health on a scale of 1 (excellent) to 5 (poor).

## Results

### Mean scores and endorsement rates

Means and standard deviations for the total score and each of the five subscales on the Spanish language QUIC (QUIC-SP) can be found in Table 3 (endorsement rates for each item are shown in S1 Table). There was a wide distribution of scores ranging from 0–32, which is similar to that reported in the English language version [1]. On average, participants endorsed having experienced 8.8 of the 38 items. Mean subscale scores ranged from 0.7 to 3.4.

**Table 3:** Means and standard deviations of the QUIC-SP score and subscale scores (*N* = 285).

	Mean/SD	Range	Internal consistency (Cronbach’s alphas)
Total Score (38 items)	8.8 (7.8)	0–32	.92
Parental monitoring and involvement (9 items)	1.9 (2.2)	0–9	.78
Parental predictability (12 items)	3.4 (3.1)	0–11	.83
Parental environment (7 items)	1.7 (1.9)	0–7	.78
Physical environment (7 items)	1.0 (1.5)	0–7	.73
Safety and security (3 items)	0.7 (1.0)	0–3	.75

### Internal reliability

There was strong internal consistency for the total score (α = 0.92) and each of the five subscale scores (α_range_ = 0.73–0.83; see Table 3). Cronbach’s alphas were consistent with those initially reported by Glynn et al. (2019) in the English language version of the QUIC.

### Associations with indicators of mental and physical health

The QUIC-SP was associated with all indicators of mental and physical health (see Table 4). Increased exposures to unpredictability in childhood assessed with the QUIC-SP predicted higher levels of anxiety, anhedonia, and depressive symptoms. Elevated QUIC-SP scores were also correlated with poorer self-reported physical functioning and global physical health.

**Table 4:** Bivariate correlations between the QUIC-SP and measures of mental and physical health.

	QUIC-SP	BDI	STAI	SHAPS	PROMIS-57	HRQOL
1. Unpredictability (QUIC-SP)	—					
2. Depression (BDI)	.42**	—				
3. Anxiety (STAI)	.36**	.75**	—			
4. Anhedonia (SHAPS)	.14*	.29**	.25**	—		
5. Physical Functioning (PROMIS-57)	-.19**	-.37**	-.29**	-.13*	—	
6. Global Health Rating (HRQOL)	.20**	.43**	.34**	.25**	-.40**	—

*QUIC-SP* Questionnaire of Unpredictability in Childhood, *BDI* Beck Depression Inventory, *STAI* State-Trait Anxiety Inventory, *SHAPS* Snaith-Hamilton Pleasure Scale, *PROMIS-57* Patient-Reported Outcomes Measurement Information System, *HRQOL* Health-Related Quality of Life

*p ≤ .05, **p ≤ .01

## Discussion

A growing body of research demonstrates that unpredictable environments in early life have a large impact on the developing brain and later emotional and cognitive functioning [1, 5–8, 14, 44]. Moreover, disproportionality in exposure to adversity in early life may play a pivotal role in explaining racial/ethnic disparities in health and development [20], yet until the present study, measures of early life unpredictability in languages other than English did not exist. The Questionnaire of Unpredictability in Childhood (QUIC) is a multifaceted self-report measure to assess early life adversity and unpredictability [1]. The current study expanded the QUIC’s utility by translating the scale into Spanish and providing evidence of internal consistency, predictive validity, and associations with important health outcomes among Spanish-speaking adults. Specifically, we showed with data from 285 adult Spanish speakers in the U.S. that there was meaningful variability in scores and strong internal consistency on the five QUIC subscales as well as the total scale score. Mirroring previous findings with the English language QUIC [1], early life unpredictability assessed with the QUIC-SP is predictive of negative mental health outcomes (depression, anxiety, and anhedonia). Further, this study adds an important novel result that early life unpredictability (as measured by the QUIC-SP) is associated with poorer physical health later in life [25, 26], strengthening the evidence that the QUIC-SP has great utility for understanding antecedents of later life health.

The development of the QUIC-SP presents several new research opportunities and opportunities to advance the inclusion of populations that may otherwise be overlooked in this critical area of research. In this study, we documented associations between the QUIC-SP and several physical and mental health outcomes, and future research can expand these findings to additional health conditions, identify possible mediators of these relations, and examine how QUIC-SP scores predict later health above and beyond other established predictors such as traumatic life events. Our sample was diverse across several demographic categories including age, gender, income, and native language (English or Spanish). The majority (78%) of our participants were born in the U.S., and future research also can expand the validity of this measure by exploring its performance and interrelations with health in Spanish-speaking populations across the globe.

Finally, we believe that understanding unpredictable environments in early life (as measured by the QUIC-SP) among Spanish-speaking populations in the U.S. will be beneficial to future researchers, given the social contextual factors that may disproportionately expose these populations to unpredictability. Over the past several years, there have been unprecedented levels of migration across the globe, including to the U.S. [45]. In 2019, Spanish was the dominant language among immigrants in the U.S. who were not primary English speakers [46]. The premigration, migration, and postmigration phases of immigration can be marked by various forms of unpredictability, such as poverty and violence in one’s home country, the migration journey, and searching for resources in a new country. The year 2022 marked the highest rates of unaccompanied child immigrants to the U.S. [47]. Upon arrival, families and children face the potential of separation and/or deportation without knowing when they may be reunited [48]. Lastly, U.S. policies on immigration are everchanging. For example, Mexican immigrants make up 89% of recipients of the Deferred Action for Childhood Arrivals (DACA) program, which allows residents brought to the U.S. as children without citizenship to apply for employment, benefits, certain schooling and schooling resources too (e.g., in state college tuition), and various funding mechanisms [46, 49]. In just the past nine years, DACA has been expanded, rescinded, and reinstated with new restrictions [50]. The program’s status continues to be in flux today. These sociopolitical considerations highlight a few of the plausible forms of unpredictability likely to be imposed on various Spanish-speaking populations in the U.S.—unpredictability that can now be captured and assessed by the QUIC-SP which includes questions about routines, parental presence and separation, parental job stability, moving, food and monetary security, and safety. As such, the QUIC-SP offers the ability to advance research and inform policy related to how unpredictability in early life impacts the health and well-being of Spanish-speaking populations.

## Supporting information

S1 Table Item-by-item endorsement rates for the QUIC-SP. (DOCX)

S1 File The QUIC-SP measure. (DOCX)
