# Supplementary material for: The QUIC-SP: A Spanish language tool assessing unpredictability in early life is linked to physical and mental health
Source: PLoS One. 2025 Jan 24;20(1):e0298296. doi: 10.1371/journal.pone.0298296 (PMC11759980; doi:10.1371/journal.pone.0298296)
Supplement: S1 Table — (DOCX) [file pone.0298296.s001.docx]

| **Table S1**  *Item-by-item endorsement rates for the QUIC-SP* |  |
| --- | --- |
| *Summary of endorsement rates* | **%** |
| Minimum | 0 |
| 25%ile | 2 |
| 50%ile | 7 |
| 75%ile | 13 |
| Maximum | 32 |
| ***Parental involvement and monitoring*** |  |
| Prior to age 12: Had a set morning routine (R) | 6 |
| Prior to age 12: Parents kept track of what I ate (R) | 18 |
| Prior to age 12: Family ate a meal together most days (R) | 31 |
| Prior to age 12: Parents made sure I got a good night’s sleep (R) | 21 |
| Prior to age 12: Had a bedtime routine (R) | 42 |
| Prior to age 12: In afterschool hours a parent knew what I was doing (R) | 14 |
| Prior to age 12: Parent regularly checked I did my homework (R) | 32 |
| Prior to age 18: Parent regularly kept track of my school progress (R) | 15 |
| Prior to age 18: Parent made time to see how I was doing (R) | 17 |
| ***Parental predictability*** |  |
| Prior to age 12: Parents often late to pick me up | 18 |
| Prior to age 12: Usually knew when parents would be home (R) | 12 |
| Prior to age 18: Parent had punishments that were unpredictable | 33 |
| Prior to age 18: Wondered if parent would come home at end of the day | 10 |
| Prior to age 18: Family planned activities to do together (R) | 30 |
| Prior to age 18: One of parents would plan something and not follow through | 28 |
| Prior to age 18: Family had holiday traditions we did every year (R) | 13 |
| Prior to age 18: Parent was disorganized | 44 |
| Prior to age 18: Parent was unpredictable | 30 |
| Prior to age 18: When parent was upset, did not know how they would act | 33 |
| Prior to age 18: Parent could go from calm to furious in instant | 44 |
| Prior to age 18: Parent could go from calm to stressed or nervous in instant | 45 |
| ***Parental environment*** |  |
| Prior to age 18: Long period of time when I didn’t see parent | 23 |
| Prior to age 18: Experienced changes in custody arrangement | 12 |
| Prior to age 18: Parent changed jobs frequently | 23 |
| Prior to age 18: Times when parent was unemployed and couldn’t find job | 32 |
| Prior to age 18: Parents had a stable relationship with each other (R) | 40 |
| Prior to age 18: Parents got divorced | 28 |
| Prior to age 18: Parent had many romantic partners | 15 |
| ***Physical environment*** |  |
| Prior to age 18: Often people coming and going in house unexpectedly | 10 |
| Prior to age 18: Moved frequently | 23 |
| Prior to age 18: Changed schools frequently | 17 |
| Prior to age 18: Changed schools mid-year | 18 |
| Prior to age 18: Lived in a clean house (R) | 10 |
| Prior to age 18: Lived in a cluttered house | 15 |
| Prior to age 18: In house things were often misplaced so could not find them | 14 |
| ***Safety and security*** |  |
| Prior to age 18: Period of time I worried would not have enough food | 18 |
| Prior to age 18: Period of time I worried family would not have enough money | 35 |
| Prior to age 18: Period of time I did not feel safe in my home | 20 |
| *Note.* (R) indicates item is reverse scored. |  |
