# Supplementary material for: The QUIC-SP: A Spanish language tool assessing unpredictability in early life is linked to physical and mental health
Source: PLoS One. 2025 Jan 24;20(1):e0298296. doi: 10.1371/journal.pone.0298296 (PMC11759980; doi:10.1371/journal.pone.0298296)
Supplement: S1 File — (DOCX) [file pone.0298296.s002.docx]

**Self-Report – Spanish**

**Questionnaire of Unpredictability in Childhood (QUIC-SP)**

**Instrucciones y Artículos:**

Este es un conjunto de preguntas sobre sus experiencias en la niñez. Cuando decimos padres, nos referimos a cualquier persona que desempeñe ese papel en su vida (por ejemplo, padres biológicos, padrastros, abuelos, padres de crianza temporal). Esto puede ser una persona, o estos pueden ser varias personas.

Por favor escriba la relación de estas personas hacia usted:

Primero, vamos a preguntarle sobre una parte específica de su niñez, que es cuando usted era menor de 12 años de edad. Estas respuestas deben ser basadas en sus propios recuerdos antes de los 12 años de edad, no en cosas que usted aprendió más tarde de sus padres u otros.

**Por favor responda a estas preguntas basándose en sus experiencias típicas o promedio.**

| **Experiencias típicas o promedio desde sus primeros recuerdos hasta la edad de 12 años…** | **Sí** | **No** |
| --- | --- | --- |
| 1. Tenía una rutina mañanera establecida en los días escolares (por ejemplo, normalmente hacía lo mismo cada día para   prepararme). | **1** | **0** |
| 1. Muchas veces mis padres llegaban tarde a recogerme (por ejemplo, de la escuela, del cuidado después de escuela o de   deportes). | **1** | **0** |
| 1. Mis padres estaban pendientes de lo que comía (por ejemplo, se aseguraban de que no me faltara una comida o intentaban asegurarse de que comiera saludable). | **1** | **0** |
| 1. La mayoría de los días comíamos en familia. | **1** | **0** |
| 1. Mis padres intentaban asegurarse de que durmiera bien por la noche (por ejemplo, tenía un horario regular de ir a dormir, mis padres revisaban que estuviera dormido). | **1** | **0** |
| 1. Tenía una rutina antes de acostarme a dormir (por ejemplo, mis padres me cobijaban, me leían un libro, yo tomaba un baño). | **1** | **0** |
| 1. Al menos uno de mis padres sabía lo que yo hacía en mis horas después de escuela o en mi tiempo libre. | **1** | **0** |
| 1. Normalmente sabía cuándo mis padres iban a estar en casa. | **1** | **0** |
| 1. Al menos uno de mis padres regularmente revisaba que yo hiciera mi tarea. | **1** | **0** |

Ahora le vamos a preguntar sobre sus experiencias desde su nacimiento a la edad de 18 años (o toda su vida, si usted es menor de 18 años de edad). Nuevamente, esto debe de basarse en sus propios recuerdos antes de los 18 años, no en cosas que usted aprendió más tarde de sus padres u otros.

**Por favor responda a estas preguntas basándose en sus experiencias típicas o experiencias promedio.**

| **Experiencias típicas o promedio desde sus primeros recuerdos hasta la edad de 18 años…** | **Sí** | **No** |
| --- | --- | --- |
| 1. Al menos uno de mis padres regularmente estaba pendiente de mi progreso en la escuela. | **1** | **0** |
| 1. Al menos uno de mis padres tenía castigos que eran impredecibles. | **1** | **0** |
| 1. Muchas veces me preguntaba si alguno de mis padres volvería a casa al final del día. | **1** | **0** |
| 1. Muchas veces había gente entrando y saliendo de mi casa que yo no me esperaba que estuvieran ahí. | **1** | **0** |
| 1. Al menos uno de mis padres hacía tiempo para ver cómo yo estaba todos los días. | **1** | **0** |
| 1. Mi familia planeaba actividades para hacer juntos. | **1** | **0** |
| 1. Al menos uno de mis padres planeaba algo para la familia pero después no llevaba el plan a cabo. | **1** | **0** |
| 1. Mi familia tenía tradiciones para los días festivos que hacíamos todos los años (por ejemplo, cocinando comida especial a cierta época del año, decorar la casa de la misma manera). | **1** | **0** |
| 1. Hubo un largo periodo de tiempo cuando no vi a uno de mis padres (por ejemplo, despliegue militar, tiempo en la cárcel,   acuerdo de mi custodia). | **1** | **0** |
| 1. Experimenté cambios en el acuerdo de mi custodia. | **1** | **0** |
| 1. Me mudé frecuentemente. | **1** | **0** |
| 1. Al menos uno de mis padres frecuentemente cambiaba de trabajo. | **1** | **0** |
| 1. Hubo tiempos cuando uno de mis padres estaba desempleado y no podía encontrar trabajo aunque él/ella quería. | **1** | **0** |
| 1. Hubo un periodo de tiempo en el que muchas veces me preocupaba de no tener suficiente comida para comer. | **1** | **0** |
| 1. Hubo un periodo de tiempo en el que muchas veces me preocupaba que mi familia no tuviera suficiente dinero para pagar por necesidades como ropa o pagos. | **1** | **0** |

| **Experiencias típicas o promedio desde sus primeros recuerdos hasta la edad de 18 años…** | **Sí** | **No** |
| --- | --- | --- |
| 1. Hubo un periodo de tiempo en el que no me sentí seguro en mi hogar. | **1** | **0** |
| 1. Cambié frecuentemente de escuelas. | **1** | **0** |
| 1. Cambié de escuelas a mitad del año. | **1** | **0** |
| 1. Mis padres tenían una relación estable entre ellos. | **1** | **0** |
| 1. Mis padres se divorciaron. | **1** | **0** |
| 1. Al menos uno de mis padres tenía muchas parejas románticas. | **1** | **0** |

Para el siguiente conjunto de preguntas, le estamos preguntando si esto es cierto para al menos uno de sus padres.

# Por favor responda a estas preguntas basándose en sus experiencias típicas o promedio.

| **Experiencias típicas o promedio desde sus primeros recuerdos hasta la edad de 18 años…** | **Sí** | **No** |
| --- | --- | --- |
| 1. Al menos uno de mis padres era desorganizado. | **1** | **0** |
| 1. Al menos uno de mis padres era impredecible. | **1** | **0** |
| 1. Para al menos uno de mis padres, cuando ellos estaban molestos, no sabía cómo ellos iban a actuar. | **1** | **0** |
| 1. Uno de mis padres podría pasar en un instante de la calma a la furia. | **1** | **0** |
| 1. Uno de mis padres podría pasar en un instante de la calma al estrés y los nervios. | **1** | **0** |

Para el siguiente conjunto de preguntas, le estamos preguntando sobre su hogar. Si usted vivió en más de un hogar, por favor responda sobre el hogar en el cual usted pasó la mayor parte del tiempo.

**Por favor responda a estas preguntas basándose en sus experiencias típicas o promedio.**

| **Experiencias típicas o promedio desde sus primeros recuerdos hasta la edad de 18 años…** | **Sí** | **No** |
| --- | --- | --- |
| 1. Vivía en una casa limpia. | **1** | **0** |
| 1. Vivía en una casa desordenada (por ejemplo, montones de cosas por todos lados). | **1** | **0** |
| 1. En mi casa, las cosas que necesitaba muchas veces no estaban en su lugar, y no las podía encontrar. | **1** | **0** |

**Scoring Information:**

The QUIC-SP Self-Report consists of an overall score and five separate subscale scores. Higher scores indicate more exposure to unpredictability in childhood. To obtain the overall score or subscale scores, reverse score select items (indicated by an R after the item number) and then calculate the sum of the items in each scale:

Parental monitoring and involvement = 1R + 3R + 4R + 5R + 6R + 7R + 9R + 10R + 14R

Parental predictability = 2 + 8R + 11 + 12 + 15R + 16 + 17R + 31 + 32 + 33 + 34 + 35

Parental environment = 18 + 19 + 21 + 22 + 28R + 29 + 30

Physical environment = 13 + 20 + 26 + 27 + 36R + 37 + 38

Safety and security = 23 + 24 + 25

Overall = Sum of all subscales

**Información de Puntaje:**

El QUIC-SP Self-Report consiste en un puntaje general y cinco subescalas de puntajes separados. Los puntajes más altos indican más exposición a la imprevisibilidad en la niñez. Para obtener el puntaje general o puntajes subescala, seleccione los artículos de puntaje inverso (indicados por una R después del número del artículo) y después calcule la suma de los artículos en cada escala:

Supervisión parental e involucración = 1R + 3R + 4R + 5R + 6R + 7R + 9R + 10R +14R

Previsibilidad parental = 2 + 8R + 11 + 12 + 15R + 16 + 17R + 31 + 32 + 33 + 34 + 35

Entorno parental = 18 + 19 + 21 + 22 + 28R + 29 + 30

Entorno físico = 13 + 20 + 26 + 27 + 36R + 37 + 38

Seguridad y protección = 23 + 24 + 25

General = Suma de todas las subescalas

**Reference/Referencia:**

Glynn, L.M., Stern, H.S., Howland, M.A., Risbrough, V.B., Baker, D.G., Nievergelt, C.M., Baram, T.Z., & Davis, E.P. Measuring novel antecedents of mental illness: The Questionnaire of Unpredictability in Childhood. *Neuropsychopharmacology*. 44, 876–882 (2019). doi: 10.1038/s41386-018-0280-9.
